# Prognosis in Chronic Diseases of the Heart

**Published:** 1870-10

**Authors:** 


Prognosis in Chronic Diseases of the Heart.—On this subject Dr. Austin Flint has written an interesting and very valuable paper, with the evident intent mainly to mollify, in some measure, the gravity of the usual prognosis in the cases referred to.

It is, he says, a popular belief that all cardiac lesions are alike dangerous: all liable to end fatally at any moment, and a patient afflicted with such disease is regarded as under sentence of death, the execution to occur without warning.

But there are lesions which are devoid of immediate danger, and sudden death from heart disease is the exception and not the rule.

Prognosis must be based on diagnosis; while by auscultation and percussion the diseases of the heart are discriminated with more precision, promptness, and positiveness than those of any other province of the nosology, a too exclusive reliance on physical signs is apt to lead to errors in prognosis, so prejudicial to the patient that it were better the information had not been obtained. This may seem strange, but it is true; it arises from want of appreciation of other circumstances than physical signs.

A patient may have the heart's action disordered, irregular, or intermittent; these symptoms may persist or recur often, and he may have dyspnœa and pain at the præcordia. General dropsy may occur, the kidneys being free from disease. With all these phenomena cardiac lesions may not exist; the disturbance may be purely functional. There may be even angina pectoris, without organic lesion. This affection may occasion death without cardiac lesion, but this must be rare. With the exception of these rare instances, if the affection of the heart be purely functional, there is no danger. The question, then, upon which prognosis hinges is: Does cardiac lesion exist? If auscultation and percussion show an absence of it, we may declare the affection functional and without danger.

Lesions of the valves which cause murmurs do not always portend immediate or even remote evil consequences. Many persons pursuing their avocations have, unconsciously, organic disease of the heart. Some of these may eventually die of this trouble, yet not a few will live to a good age, and die of some other disease. But let such an one be informed of his malady, and he has received a blow from which he will never recover; he looks upon himself as doomed. The depressing effect of the diagnosis shortens his life. "I have records of cases in which organic endocardial murmurs existed from ten to thirty years ago, the persons now living, and exempt from ailments referrable to diseases of the heart."

The intensity and quality of heart murmurs are not of much account in judging of the importance of valvular lesions. A loud, rough, or musical murmur does not necessarily indicate a more grave lesion than one that is soft, feeble, and blowing.

Barring the accident of embolism, in valvular lesions producing murmur, there is no danger so long as the heart is not enlarged; for valvular lesions cause enlargement of the heart before any other effect that jeopardizes life. Moreover, in most cases, enlargement of the heart from valvular lesions is produced slowly, the ulterior effects being proportionately distant.

Hypertrophy of the heart, as a result of obstruction to the blood-current, or regurgitation, or both, is a conservative provision. The increased muscular power makes amends for the disturbance of circulation, and prevents evils which would otherwise ensue. A patient is comparatively safe even though the heart be greatly enlarged, provided this be from muscular growth or hypertrophy. There is, however, nothing conservative in dilatation, but the opposite. In proportion to the dilatation is the ability of affective contraction impaired; it increases the labor of the contraction by allowing a larger accumulation of blood. It is by means of this weakness that valvular lesions lead to remote evils—systemic congestion and general dropsy. Hence the necessity to determine whether an enlargement is a dilatation or hypertrophy.

While hypertrophy compensates for valvular lesion, this in turn sometimes may be conservative as regards hypertrophy. Certain evils, liable to occur from hypertrophy of the left ventricle if the valves be sound, are warded off by co-existing valvular lesions.

Statistics show that there is more danger of sudden death from distension with blood, and paralysis of the left ventricle as a result of aortic obstruction or regurgitant lesions, when these exist alone, than when associated with mitral lesions.

Cases occur in which, with organic disease of little consequence, functional disturbance is induced by other causes. This is very apt to be imputed to the organic lesion, and a needlessly grave prognosis be given.

There is a vast difference among patients in the toleration of chronic heart disease; the power to withstand its effects is in proportion as the other functions of the body are healthfully performed; this relates more especially to the whole function of nutrition.

In the majority of cases of chronic heart disease, the cardiac lesion is not exclusively or directly the cause of death. Most patients perish from intercurrent affections, dependent upon, or accidentally associated with, the heart lesion. Of the cases where cardiac lesions are fatal, sudden death occurs in a very small proportion.

Of valvular lesions, those which occasion aortic regurgitation involve the greatest liability to sudden death. The immediate cause of death is paralysis of the left ventricle from over-distension.

Fatty degeneration, with valvular lesion and dilatation increases the liability to sudden death; but it is not easy to determine this combination during life.

There is danger of sudden death when angina pectoris is associated with organic disease of the heart, and most when this latter is aortic regurgitation, the mitral valve being sound; and association with fatty degeneration increases the danger. In angina there is little or no danger, unless the patient have a sense of impending death and feels the necessity of perfect quietude.—*New York Journal of Medicine.*

